# Effectiveness of exercise in reducing cerebral stroke risk factors: A systematic review and meta-analysis

**DOI:** 10.1097/MD.0000000000031861

**Published:** 2022-11-11

**Authors:** Jie Men, Wenjuan Wang, Jian Zhao, Jie Wen, Qingqing Hao, Shufeng Li, Shuangling Zou

**Affiliations:** a Department of Medical Laboratory Science, Fengyang College, Shanxi Medical University, Shanxi, China.

**Keywords:** carotid atherosclerosis, carotid intima-media thickness, cerebral stroke, exercise

## Abstract

This study aimed to explore the relationship between exercise and cerebral stroke and provide evidence for the prevention of cerebral stroke.

**Materials/Methods:**

All clinical trials of exercise intervention for atherosclerosis were systematically reviewed. Five major databases were searched to retrieve relevant studies from their inception to May 2022. According to the magnitude of heterogeneity, the random and fixed-effect models were used to test reasonably.

**Results:**

According to the inclusion and exclusion criteria, 1341 articles were screened and 13 articles involving 825 patients were identified. The result showed that in the randomized controlled trials carotid intima-media thickness index was lower in the exercise group (−0.04 mm, 95% confidence interval: −0.07 to −0.01). All were statistically significant (*P* < .005) and subgroup analysis showed that the intervention period and paper quality are sources of heterogeneity.

**Conclusions:**

The results of this systematic review suggest that exercise is associated with a slow increase in carotid intima-media thickness, which may provide evidence that exercise helps reduce cerebral stroke.

## 1. Introduction

The Global Burden of Diseases, Injuries, and Risk Factors Study in 2017 showed that cerebral stroke (also known as stroke) is the second leading cause of death and disability.^[1]^ The data showed that from 1990 to 2019, the absolute number of global cerebral stroke events, the prevalence of cerebral stroke and stroke-related deaths increased by 70.0%, 85.0% and 43.0%, respectively.^[2]^ With its high rate of morbidity and mortality, cerebral stroke tends to induce sequelae or lead to severe complications and seriously affects the quality of life of patients and causes great harm to individuals and society. It is highly associated with hypertension, dyslipidemia, diabetes, smoking, low physical activity, obesity, atherosclerosis (AS) and other factors.^[3]^ Studies have shown that people with type 2 diabetes have nearly double the risk of cerebral stroke compared with people without diabetes.^[4,5]^ Meanwhile, meta-analyses of prospective studies have explained that increasing BMI is independently associated with an increased risk of cerebral stroke.^[6]^ Remarkably, the 2 fastest-growing risk factors for stroke are overweight and obesity.^[2]^ In cerebral stroke with obesity, the susceptibility to ischemic brain damage is significantly increased.^[7]^ In addition, AS is also the most common cause of cerebral stroke.^[8]^

Studies have shown that the incidence of carotid AS and cerebral stroke is closely related to the severity of the disease,^[9]^ approximately 18% to 25% is attributed to AS in the carotid artery.^[10]^ We can know that delaying the progression of AS has become 1 of the main methods to prevent cerebral stroke. And researches have indicated that carotid intima-media thickness (CIMT) is not only a significant indicator of subclinical AS but also a strong predictor of future cerebral stroke.^[11]^ Scientific studies have proved that if the difference in CIMT is 0.1mm, the risk of stroke increases by 13% to 18%.^[12]^ Therefore, stroke can be effectively prevented by slowing down the rate of change in thickness rate.

At present, the standard clinical treatment for stroke is thrombolysis and anticoagulation therapy, but the window period of thrombolytic therapy is very short (less than 4.5 hours),^[13]^ and anticoagulation therapy drugs can cause abnormal coagulation function and increase the risk of bleeding and stroke again.^[14]^ With the progress of stroke research, the clinical focus has gradually shifted from treatment to prevention. Increasing evidence indicated that regular exercise is beneficial to decrease obesity^[15]^ and diabetes,^[16]^ declining the CIMT^[17]^ and preventing the occurrence of AS,^[18]^ thereby reducing the risk of stroke.^[19]^ Compared with medical and surgical treatments, exercise is a simple, non-drug and low-cost alternative to drug and surgical treatment, and it can effectively improve the patient’s status, protect against or even delay the onset of complications. However, there is some controversy about the reduction of CIMT by exercise,^[20,21]^ and the effectiveness of exercise remains to be proven. Therefore, this study used a meta-analysis to summarize the efficacy of different exercise programs in reducing stroke risk factors, aiming to provide corresponding evidence-based medical evidence for the development of stroke prevention programs.

## 2. Materials and methods

This review follows the Preferred Reporting Items for Systematic Review and Meta-Analyses guidelines,^[22]^ whose consent from the Human Ethics Committee is not required, and merely provides a systematic summary and analysis of relevant literature. The search and method were pre-specified, and we have pre-registered with PROSPERO (10.37766/inplasy2022.9.0078).

### 2.1. Literature retrieval strategy

Most of the related articles were found through a comprehensive search. Additional articles were found through citations and references.

Five databases were searched from inception to May 2022: PubMed, Cochrane Library, Web of Science, Embase and Scopus. Search for the keywords “Cerebral Stroke,” “CIMT,” “Exercise” and “Carotid AS.”

Two authors (Jian Zhao and Jie Wen) excluded duplicated studies, independently selected articles according to established rules, assessed whether the article met the inclusion and exclusion criteria and collected data. Two people would adopt a unified standard of judgment if there were questions to find another author (Wenjuan Wang) for discussion. To further identify relevant studies, we conducted a secondary search of references for all articles.

### 2.2. Inclusion and exclusion criteria

#### 2.2.1. Inclusion criteria.

##### 2.2.1.1. Participants.

Potential high-risk groups for stroke (overweight/obesity, diabetes) and no contraindications to exercise.

##### 2.2.1.2. Interventions.

No special restrictions on the exercise intervention programs (form, intensity, frequency, time, etc).

##### 2.2.1.3. Comparisons.

Randomized controlled experiments with no exercise intervention in the control group.

##### 2.2.1.4. Outcomes

Need to include the outcome of the CIMT.

##### 2.2.1.5. Study

Literature published in English.

#### 2.2.2. Exclusion criteria.

- (1) Diagnosed with a stroke or stroke rehabilitation.
- (2) Incomplete data and lack of necessary description of the exercise intervention plan.
- (3) Review, Animal Experiments and Case Reports.

### 2.3. Data synthesis

The analysis used Review Manager 5.4 (Cochrane Collaboration, Oxford, UK) and Stata 15. The mean difference and the standard deviation were used as the effective indices for the measurement data, and the point estimate and 95% confidence interval were given for each effect. *I^2^* was used to quantitatively determine the size of heterogeneity; *I^2^* values greater than 50% were considered indicative of heterogeneity in our study. When *I^2^* was greater than 50%, the random-effects model was used instead of the fixed-effects model. Otherwise, the opposite is true.^[23]^

## 3. Results

### 3.1. Study selection

After an initial search of 1341 articles, 1081 articles were excluded by removing duplicate documents and reading the title and abstract. Finally, after reading the full text, we identified 13 articles that met the requirements. Many forms of exercise were included and statistical analyses were performed on this basis. The content of the literature selection process and the results are shown in the Guidelines Flow Diagram.

### 3.2. Qualitative study characteristics

Table 1 lists the characteristics of the included articles (study, age, sample size, exercise form, etc). Eight hundred twenty-five participants were included in the study for analysis, including 438 in the exercise group and 387 in the control group (Table 1).

**Table 1:** Characteristics of the included studies.

Study	P	Number		Age		Intervention		Duration		
		E	C	E	C	E	C			
Park, J.H et al, 2015^[24]^	Obesity	10	10	70.70 ± 0.70	71.30 ± 0.60	Aerobic, resistance exercise, traditional Korean dance, 3 times/wk	No exercise	3 mo		
Park, J et al (1), 2017^[25]^	Obesity	25	25	73.50 ± 7.10	74.70 ± 5.10	Consisting of resistance, aerobic training 50–80 min, 5 times/wk	No exercise	6 mo		
Meyer et al, 2006^[26]^	Obesity	33	34	13.70 ± 2.10	14.10 ± 2.40	Exercise 60 min, 3 times/wk	No exercise	6 mo		
Park, J et al (2), 2017^[27]^	Obesity	21	20	70.40 ± 4.50	68.40 ± 2.60	Aerobic, resistance exercise, 40–80 min, 5 times/wk	No exercise	6 mo		
Byrkjeland et al, 2016^[28]^	Diabetes	61	62	63.50 ± 8.00	63.20 ± 7.20	Aerobic, resistance training 150 min	No exercise	12 mo		
Rahbar et al, 2017^[21]^	Diabetes	13	15	48.31 ± 5.02	48.60 ± 4.80	Aerobic exercise 30 min, 3 times/wk	No exercise	2 mo		
Hetherington et al, 2020^[29]^	Diabetes	41	22	58.80 ± 8.10	60.80 ± 7.50	MCT with RT, HIIT with RT	No exercise	12 mo		
Liu et al, 2013^[30]^	Diabetes	40	21	49.80 ± 4.80	49.80 ± 4.80	Moderate intensity walking exercise, walking plus resistance exercise, 60 min, 4 times/wk	No exercise	6 mo		
Ghardashi et al, 2020^[20]^	Diabetes	30	29	55.10 ± 6.07	54.10 ± 5.68	85%–90% HRmax HIIT, 45%–50% HRmax HIIT, 3 times/wk	no exercise	3 mo		
Farahati et al (1), 2021^[31]^	Obesity	11	9	43.37 ± 3.29	44.22 ± 3.63	60–70% HRmax resistance Exercise 47 min	No exercise	3 mo		
Farahati et al (2), 2021^[31]^	Obesity	10	9	43.37 ± 3.29	44.22 ± 3.63	85%–95% HRmax HIIT	No exercise	3 mo		
Kim et al, 2006^[32]^	Diabetes	32	26	55.00 ± 8.10	53.80 ± 9.00	Activity of moderate intensity 150 min/wk	No exercise	6 mo		
Magalhaes et al (1), 2019^[33]^	Diabetes	16	22	59.70 ± 7.00	60.80 ± 7.50	MCT with RT, 3times/w	No exercise	12 mo		
Magalhaes et al (2), 2019^[33]^	Diabetes	13	22	59.70 ± 7.00	60.80 ± 7.50	HIIT with RT, 3 times/wk	No exercise	12 mo		
Koeder et al, 2021^[34]^	Obesity	82		61		59.4 ± 1.0	54.7 ± 1.4	a healthy lifestyle program	no exercise	6 mo

C = control group, E = excerise group, HIIT = high-intensity interval training, MCT = moderate continuous training, P = participants, RT = resistance training, Wk = week

### 3.3. The study bias risk assessment

**Figure 1.**
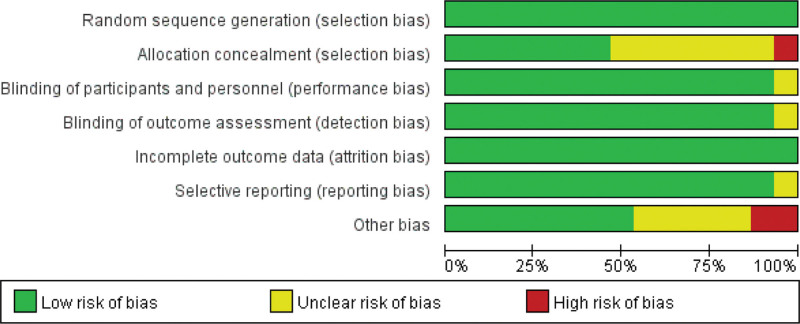
Risk of bias graph: judgments about each risk of bias item presented across all included trials.

**Figure 2.**
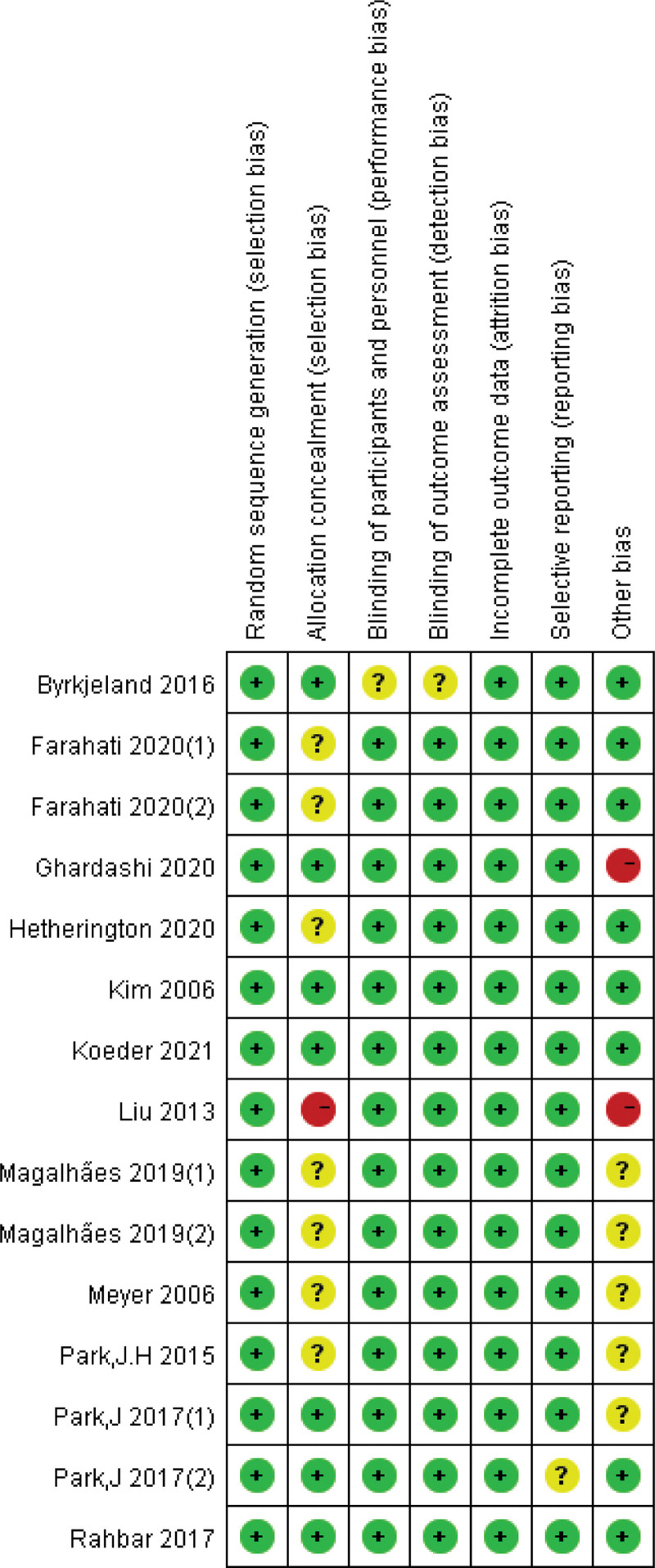
Risk of bias summary: judgments about each risk of bias item for each included trial.

Two authors (Jie Men and Jian Zhao) independently evaluated and cross-checked the results using the RCT risk of bias assessment tool recommended by the Cochrane5.1.0 bias assessment tool, as shown in Figures 1 and 2.

## 4. Meta-analysis results

### 4.1. Meta-analysis of experimental studies

**Figure 3.**
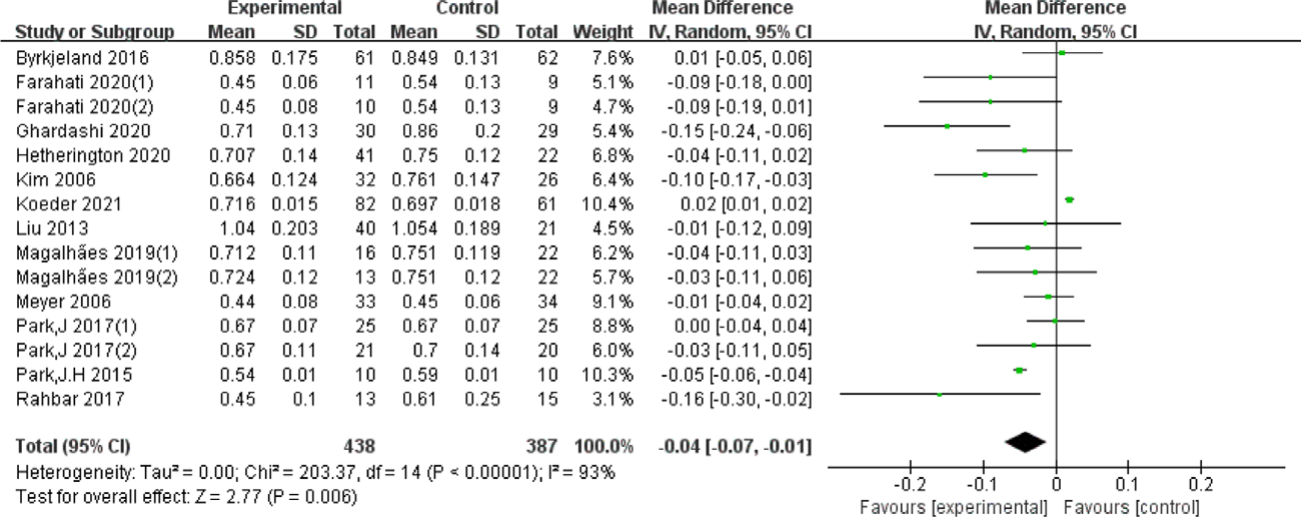
Forest plot of meta-analysis on the effect of exercise on CIMT. CI = confidence interval, CIMT = carotid intima-media thickness, SD = standard deviation.

We included 13 RCTs,^[20,21,24–34]^ and the combined results suggested potentially significant and statistically significant differences between exercise and carotid artery intima-media thickness (Fig. 3).

#### 4.1.1. Heterogeneity analysis of experimental studies.

**Figure 4.**
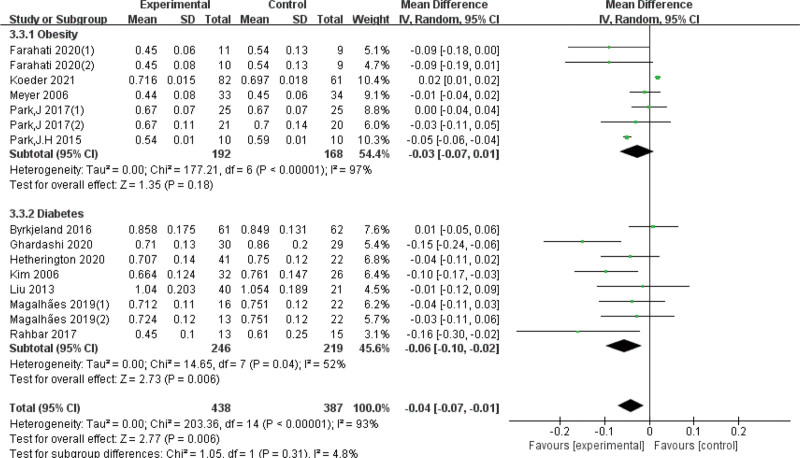
Forest plot of subgroup analysis on the effect of participants. CI = confidence interval, SD = standard deviation.

**Figure 5.**
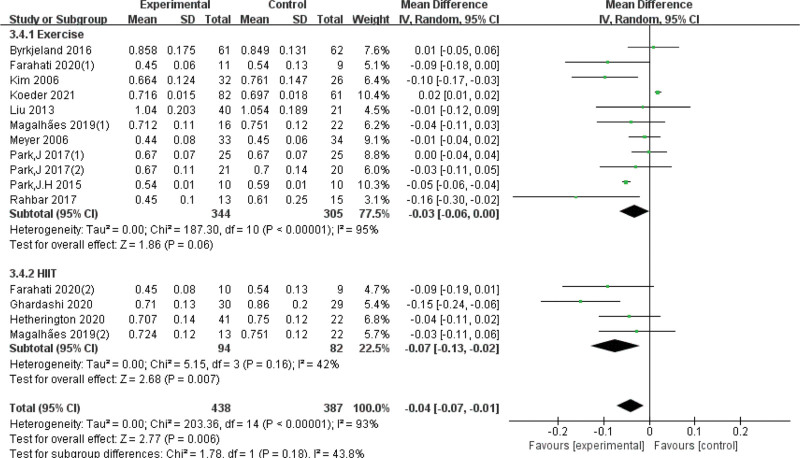
Forest plot of subgroup analysis on the effect of exercise intensity. CI = confidence interval, SD = standard deviation.

**Figure 6.**
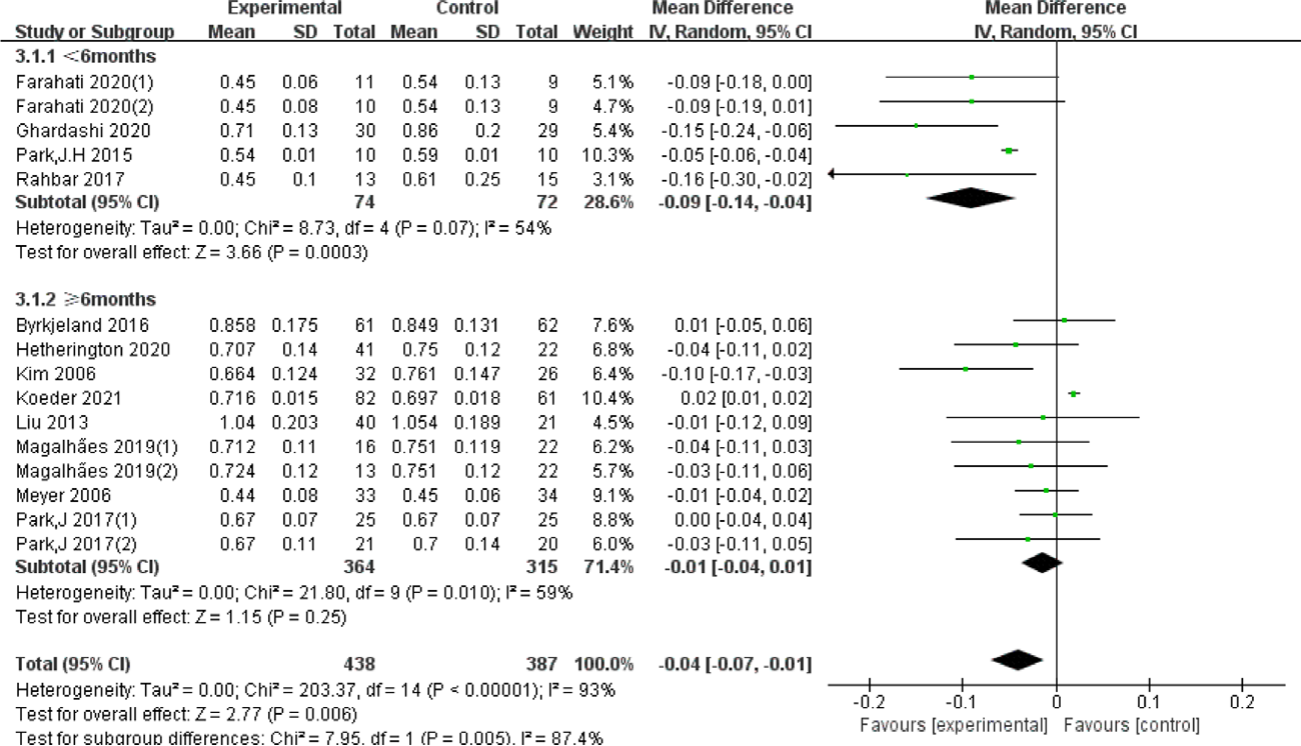
Forest plot of subgroup analysis on the effect of the intervention time. CI = confidence interval, SD = standard deviation.

**Figure 7.**
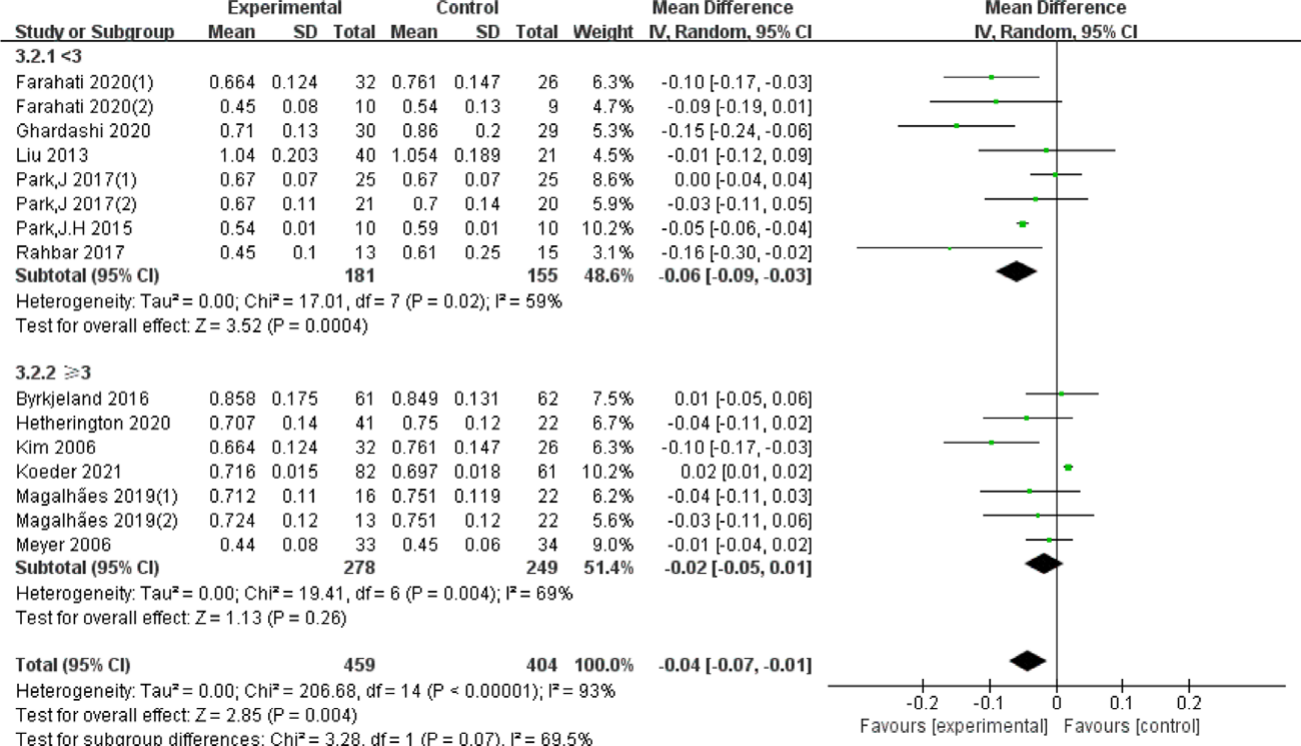
Forest plot of subgroup analysis on the effect of the article impact factor. CI = confidence interval, SD = standard deviation.

Due to the high heterogeneity, subgroup analyses were conducted based on exercise duration. The differences were statistically significant in the subgroup of exercise < 6 months (*I*^*2* ^= 54%, *P* < .01) and the subgroup of exercise ≥ 6 months (*I*^*2 *^= 59%, *P* < .01), we also analyzed the quality of the articles, it clear that heterogeneity has gone down. In addition, subgroup analyses of intervention intensity and enrolled subjects showed that neither was the source of heterogeneity (Table 2, Figs. 4–7).

**Table 2:** Subgroup analysis results table.

Outcome measures	Subgroup		The Number of studies	Pooled estimate [SMD/MD (95% CI)]	*P* value	*I^2^*(%)	Test for subgroup differences
CIMT	Exercise duration	<6 mo	5	−0.09 [−0.14, −0.04]	*P* = .07	54	*P* = .005*
		≥6 mo	10	−0.01 [−0.04, 0.01]	*P* = .01	59	
	The quality of the articles	<3	8	−0.06 [−0.09 ,−0.03]	*P* = .02	59	*P* = .07
		≥3	7	−0.02 [−0.05, 0.01]	*P* = .004	69	
	Intervention intensity	Exercise	11	−0.03 [−0.06, 0.00]	*P* < .001	95	*P* = .18
		HIIT	4	−0.07 [−0.13, −0.02]	*P* = .16	42	
	Participants	Obesity	7	−0.03 [−0.07, 0.01]	*P* < .001	97	*P* = .31
		Diabetes	8	−0.06 [−0.10, −0.02]	*P* = .04	52	

CIMT = carotid intima-media thickness, HIIT = high-intensity interval training.

* =*P* < .05.

#### 4.1.2. Sensitivity analysis of descriptive research.^[35]^

**Figure 8.**
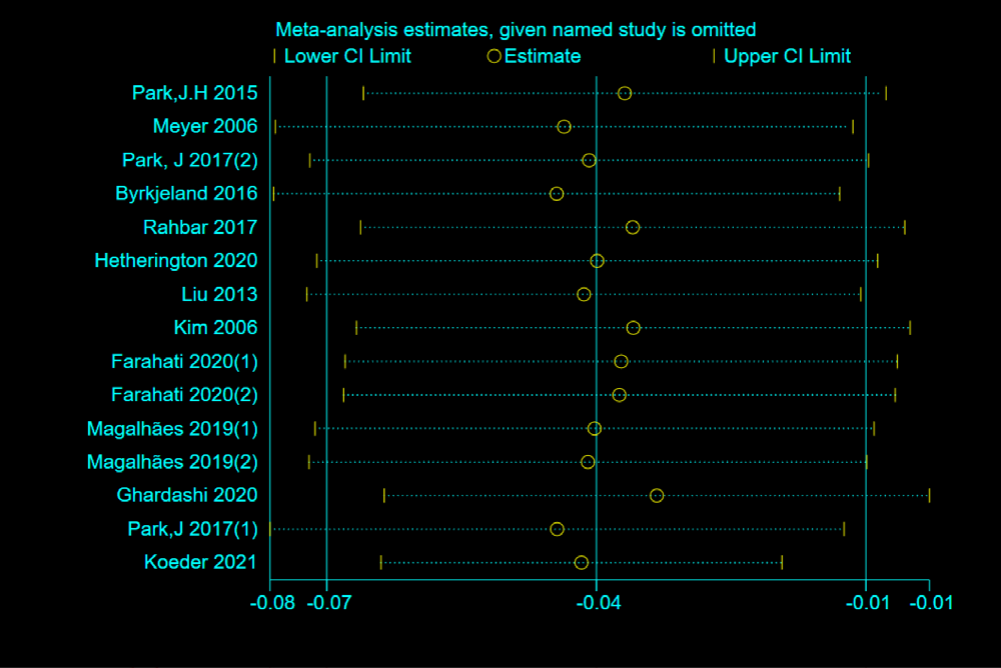
The sensitivity analysis of the included studies.

The result of the sensitivity analysis was negative (Fig. 8), indicating that the effect size synthesized by deleting literature was consistent with the total effect size. This process confirms the stability of the overall results.

#### 4.1.3. Publication bias test.

**Figure 9.**
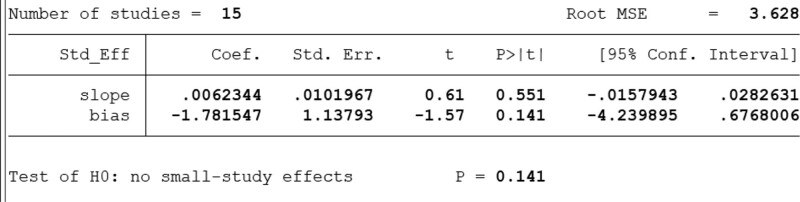
The results of Egger’s published bias.

There was no apparent publication bias in this study (*P *> .05) (Fig. 9).

## 5. Discussion

This systematic review synthesized 13 eligible randomized controlled trials^[20,21,24–34]^ to explore the clinical effect of exercise in improving CIMT for cerebral stroke prevention. We found that exercise could significantly reduce the CIMT, which is crucial for stroke prevention. Exercise intervention programs are also critical factors. The results of the CIMT measurements have substantial heterogeneity, which is caused by various factors. First, our meta-analysis included overweight, obese, and diabetic populations, who are at increased risk of stroke due to insufficient physical activity, which predisposes them to dyslipidemia and AS.^[35]^ Second, participants varied in the intensity of exercise interventions, with 9 studies^[21,24–28,30,32,34]^ using moderate-intensity exercise, 4 studies^[20,29,31,33]^ using high-intensity exercise. It is generally believed that high-intensity exercise will produce more benefits for the body,^[36]^ but moderate-intensity exercise is usually recommended for people with obesity and diabetes.^[37]^ Third, the duration of intervention in the study ranged from 8 weeks to 12 months, and it is generally believed that a longer exercise intervention time may lead to a better prevention impact. Fourth, there were differences in the quality of the included studies. The impact factors of the 13 included studies range from 1 to 28. Generally speaking, the higher the literature impact factor, the more rigorous the research plan. We conducted subgroup analyses and found that the reason for heterogeneity was the duration of the exercise intervention rather than the participants, exercise intensity, or paper quality. An exercise intervention duration of less than 6 months had a more significant effect on CIMT, which suggests that a longer duration of exercise intervention did not appear to bring more benefits. We speculate that this difference may be caused by the diminished sensitivity of prolonged exercise intervention to slowing the CIMT. Furthermore, exercise was more effective in people with diabetes, and high-intensity interval training seemed to have a more significant effect than moderate continuous training, which lead to the conclusion that patients with diabetes are more sensitive to prolonged high-intensity interval training intervention. Nonetheless, the results of the subgroup analysis may also be due to differences in baseline levels and sensitivity to exercise among the participants.

Globally, there are many studies on the improvement of CIMT by exercise, mainly the role of exercise in the treatment of diseases related to media thickness, such as stroke, AS, cardiovascular and cerebrovascular diseases. However, systematic reviews have assessed the role of exercise in improving CIMT in stroke prevention. This systematic review showed that exercise significantly reduces CIMT and has a positive effect on stroke prevention, similar to the result reported in a systematic review by Garcia-Hermoso et al^[38]^ In a prospective trial lasting 6.5 years, participants with regular physical activity had lower mean progression rates of maximum CIMT than sedentary participants, which may translate to differences in cardiovascular risk.^[39]^ In contrast, a systematic review by Lauche et al^[40]^ reported that the evidence did not support the role of exercise in preventing stroke compared with the control group. It should be noted that the exercise methods are Tai Chi and Qigong, focusing more on breathing and meditation exercises, which seem to be less intense but have a significant effect on improving blood pressure and seems to have some potential for preventing stroke. In addition, the studies included Byrkjeland et al^[28]^ and Liu et al^[30]^ reported that exercise did not improve CIMT compared with controls. Both studies used aerobic exercise combined with resistance training. This seems to affect the effect of exercise intervention, which was consistent with the conclusion of Kadoglou et al,^[41]^ who investigated the relationship between different exercise modes and the progression of early carotid AS, and found that only aerobic training can improve the CIMT of patients, while Resistance training or the combination of resistance training and aerobic exercise had no significant effect. In addition, the additional benefits of exercise intervention on CIMT and AS may be attenuated when participants are on statins.^[42]^ At the same time, it is worth noting that exercise reduces the arterial media thickness in patients with early AS, whereas the effect of exercise on vascular lesions in patients with advanced AS is weakened. The presence of plaque may interfere with endothelial function and weaken the impact of exercise. Given that CIMT is highly correlated with stroke,^[43]^ most of the reports that hold the opposite conclusion to ours are based on a randomized controlled trial. However, we are a systematic review based on multi-sample randomized controlled trials, which further confirms the expert consensus that exercise interventions improve vascular endothelial function, delay plaque progression and reduce the risk of stroke.

This meta-analysis was carried out in strict accordance with the requirements, but there are still some limitations: First, although this review has a rigorous search strategy, due to limited conditions, only literature published in English may be searched, and a small amount of published literature may still be missing, which may lead to certain publication bias. Second, the heterogeneity of the study results was relatively high, and although the source of heterogeneity was identified, the results of this systematic review should be interpreted with caution. Third, the 2 studies included in our meta-analysis were inconsistent with the meta-analysis results, and no definitive clinical recommendations could be made. We look forward to more relevant studies to further expand the content, and improve the reliability of the results. Fourth, although exercise has a positive effect on cerebral stroke prevention, there are no specific exercise programs.

## 6. Conclusions

Based on the results of this meta-analysis, we can conclude that exercise significantly reduces the CIMT and has the potential to prevent cerebral stroke. Considering its low cost and few side effects, exercise should be included as a preferred measure for stroke prevention. However, it remains unclear whether exercise has a graded effect on the progression of CIMT and the long-term risk of cerebrovascular events. In the future, more well-designed large-sample randomized controlled trials and long-term prospective studies are needed to clarify the preventive effects.

## Author contributions

Wenjuan Wang contributed to study design, manuscript preparation, and data analysis. Jian Zhao and Jie Men contributed to the data collection and critical revision of the essential intellectual content of the manuscript. All authors have contributed to this article.

**Data curation:** Jian Zhao, Jie Wen, Shuangling Zou.

**Funding acquisition:** Qingqing Hao.

**Investigation:** Wenjuan Wang, Shufeng Li.

**Methodology:** Wenjuan Wang, Shuangling Zou.

**Supervision:** Jie Men, Qingqing Hao.

**Writing – original draft:** Wenjuan Wang.

## Correction

Shuangling Zou has been added to the author list for this article.
